# Finerenone: A Third-Generation MRA and Its Impact on Cardiovascular Health—Insights from Randomized Controlled Trials

**DOI:** 10.3390/jcm13216398

**Published:** 2024-10-25

**Authors:** Michael Sabina, Jennifer Trube, Shrinand Shah, Andrew Lurie, Mason Grimm, Anas Bizanti

**Affiliations:** Lakeland Regional Health Medical Center, Lakeland, FL 33805, USA; jennifer.trube@mylrh.org (J.T.); shrinand.shah@mylrh.org (S.S.); andrew.lurie@mylrh.org (A.L.); mason.grimm@mylrh.org (M.G.); anas.bizanti@mylrh.org (A.B.)

**Keywords:** finerenone, heart failure, steroidal mineralocorticoid receptor antagonist, spironolactone, eplerenone, systematic review

## Abstract

**Introduction:** Finerenone, a third-generation non-steroidal mineralocorticoid receptor antagonist (MRA), offers a targeted approach to managing cardiovascular outcomes, particularly in patients with chronic kidney disease (CKD) and type 2 diabetes (T2D). Unlike traditional MRAs such as spironolactone and eplerenone, which can cause off-target hormonal side effects and hyperkalemia, Finerenone selectively binds to mineralocorticoid receptors, reducing these risks. Recent randomized controlled trials have demonstrated Finerenone’s potential to improve cardiovascular outcomes, making it a promising alternative in the management of heart failure and other cardiovascular conditions associated with CKD and T2D. **Methods:** We conducted a scoping review using PRISMA guidelines. A search for “Finerenone” in the PubMed, Embase, and Cochrane Library databases included randomized controlled trials (RCTs), post hoc analyses, and relevant meta-analyses on cardiovascular outcomes. Data were synthesized narratively, assessing study quality through strengths and limitations. **Discussion:** Finerenone has shown significant benefits and a superior safety profile compared with traditional MRAs like spironolactone and eplerenone in managing CKD, T2D, and heart failure. It effectively reduces cardiovascular and renal events while minimizing risks such as hyperkalemia and hormonal side effects associated with steroidal MRAs. Future studies, including the REDEFINE-HF, FINALITY-HF, and CONFIRMATION-HF trials, will further explore Finerenone’s potential across diverse heart failure phenotypes, including its role in heart failure with mildly reduced and preserved ejection fractions, potentially establishing it as a cornerstone therapy in heart failure management. **Conclusions:** Finerenone represents a significant advancement in MRA therapy, offering enhanced safety and efficacy in managing cardiovascular outcomes in CKD and T2D patients. The current evidence supports its use as a promising alternative to traditional MRAs, particularly in patients intolerant to steroidal MRAs. Further trials are needed to fully establish its potential across diverse patient populations, including those with varying heart failure phenotypes.

## 1. Introduction

Mineralocorticoid receptor antagonists (MRAs) like spironolactone and eplerenone are well-established therapies for managing heart failure with reduced ejection fraction (HFrEF), as recommended by European and US guidelines for symptomatic patients with this condition [1,2]. These medications are effective due to their ability to block the harmful effects of aldosterone on the cardiovascular system. Despite their benefits, the use of these steroidal MRAs is often limited by their associated risks, including hyperkalemia and off-target effects on androgen and progesterone receptors, which can lead to undesirable side effects such as gynecomastia and hormonal imbalances [3,4]. These challenges are particularly pronounced in patients with comorbid conditions like chronic kidney disease (CKD) and type 2 diabetes (T2D), where the risk of adverse events is heightened.

Finerenone, a third-generation non-steroidal MRA, represents a significant advancement in this therapeutic class. By selectively binding to the mineralocorticoid receptor without affecting other steroid receptors, Finerenone offers a more targeted approach, potentially reducing the incidence of hyperkalemia and other side effects seen with traditional MRAs. Recent randomized controlled trials have explored its efficacy in improving cardiovascular outcomes among patients with CKD and T2D, highlighting its role as a promising alternative in the management of heart failure and related conditions. This review aims to synthesize the current evidence on Finerenone’s cardiovascular benefits, providing a comprehensive overview of its potential role in modern clinical practice.

## 2. Methods

**Figure 1:**
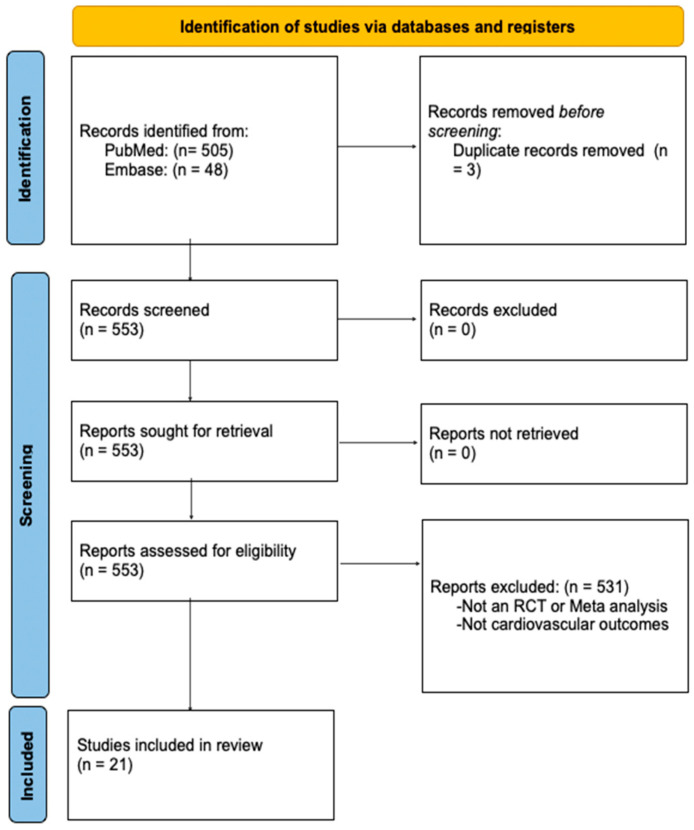
PRISMA flow diagram.

This scoping review aimed to map the available evidence on Finerenone’s impact on cardiovascular health by including randomized controlled trials (RCTs), post hoc analyses, and relevant meta-analyses. We conducted a comprehensive search using the term “Finerenone” without time constraints across the PubMed, Embase, and Cochrane Library databases. We followed PRISMA guidelines and the methodological framework by Arksey and O’Malley [5,6]. RCTs and post hoc analyses were included if they assessed Finerenone’s cardiovascular effects. Meta-analyses were considered if they included eligible RCTs and reported cardiovascular outcomes. No language restrictions were applied. Data were charted to capture the study characteristics, interventions, and key outcomes and synthesized narratively. The quality of the studies was assessed through a discussion of their strengths and limitations rather than through formal quantitative analyses such as risk of bias or heterogeneity assessments. This approach provided an overview of the evidence landscape relevant to Finerenone and cardiovascular health (Figure 1).

## 3. Results

### 3.1. Mineralocorticoid Receptor Antagonists

#### 3.1.1. Spironolactone

**Figure 2:**
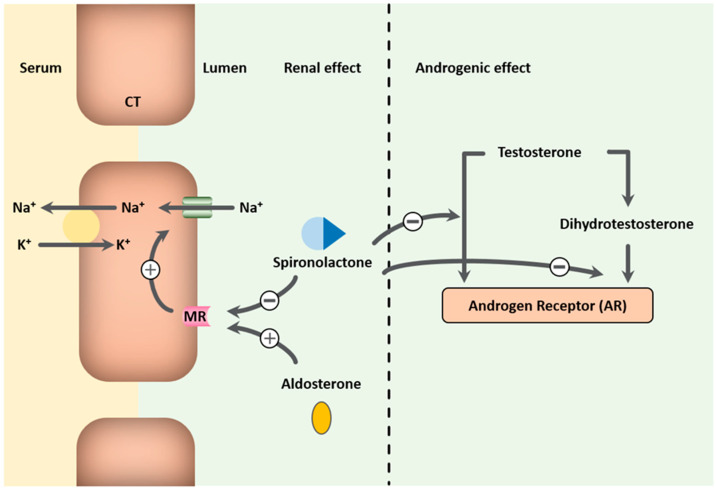
Spironolactone’s action on mineralocorticoid receptors and androgen receptors. Legend: MR = Mineralocorticoid receptor.

MRAs exhibit diuretic and antihypertensive effects by competitively inhibiting aldosterone at mineralocorticoid receptors in the heart, kidneys, and blood vessels. Spironolactone is a nonselective MRA that not only binds to mineralocorticoid receptors but also to androgen and progesterone receptors (Figure 2). Aldosterone, part of the renin-angiotensin–aldosterone system, acts on the distal tubules and collecting ducts, promoting sodium reabsorption and potassium secretion, and increasing vascular stiffness, cardiac inflammation, fibrosis, and remodeling. Blocking these actions helps reduce the harmful effects of aldosterone that can worsen HFrEF [7,8]. MRAs also help maintain serum potassium levels by decreasing urinary potassium loss, lowering the risk of hypokalemia and related arrhythmias associated with non-potassium-sparing diuretics [9,10,11]. They are primarily used in treating heart failure, particularly HFrEF, and have shown benefits in reducing morbidity and mortality when used alongside standard therapies like angiotensin-converting enzyme inhibitors (ACEis) or angiotensin receptor blockers (ARBs) [10,11,12]. The 2017 ACC heart failure guidelines recommend spironolactone for NYHA class II-IV HFrEF patients with a creatinine clearance above 30 mL/min and serum potassium below 5 mEq/L, and they suggest potential benefits for select heart failure with preserved ejection fraction (HFpEF) patients in reducing hospitalizations [1]. However, the use of spironolactone does not come without side effects. Its use can lead to hyperkalemia and impaired renal function, requiring close monitoring of serum potassium levels and kidney function [13]. Despite this, the Effect of Spironolactone on Morbidity and Mortality in Patients with Severe Heart Failure (RALES) trial, the Eplerenone, a Selective Aldosterone Blocker, in Patients with Left Ventricular Dysfunction after Myocardial Infarction (EPHESUS) trial, and the Eplerenone in Patients with Systolic Heart Failure and Mild Symptoms (EMPHASIS-HF) trial have demonstrated the efficacy of MRAs in HFrEF patients being treated with ACE inhibitors. This is likely due to their ability to address aldosterone escape and to reduce aldosterone effects that ACE inhibitors alone cannot manage [9,11,14,15]. Furthermore, spironolactone exhibits anti-androgenic effects due to its activity as a progesterone receptor agonist and androgen receptor antagonist, which can result in gynecomastia, breast tenderness, menstrual irregularities, impotence, and decreased libido, especially at higher doses [3,4]. Hence, it is an off-label, non-Food and Drug Administration (FDA)-approved medication for the treatment of hirsutism and acne vulgaris [16].

#### 3.1.2. Eplerenone

In contrast, eplerenone, a second-generation aldosterone antagonist, is more selective for the mineralocorticoid receptor than spironolactone, with a significantly lower affinity for glucocorticoid, progesterone, and androgen receptors, offering a 100- to 1000-fold reduction in binding to these receptors [17]. It is FDA-approved for improving survival in stable HFrEF patients with congestive heart failure (CHF) post-myocardial infarction, which is supported by findings from the EPHESUS and EMPHASIS-HF trials [10,11]. Eplerenone is also approved for treating hypertension, alone or in combination, particularly when patients experience adverse effects from spironolactone [18]. A multicenter trial showed that eplerenone had fewer endocrine side effects and was better tolerated than spironolactone, although it was less effective in lowering diastolic blood pressure [19]. Thus, starting with spironolactone and switching to eplerenone if side effects arise is a practical approach.

#### 3.1.3. Finerenone

**Figure 3:**
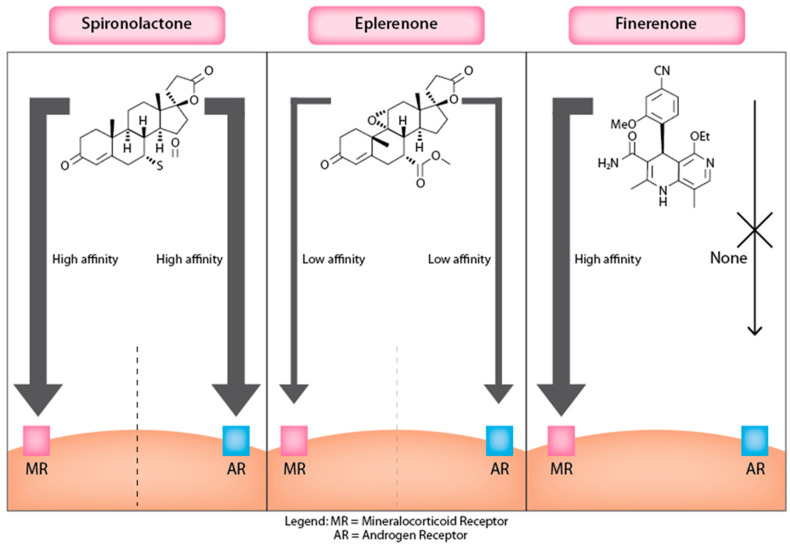
MRA’s action on mineralocorticoid and androgen receptors.

Finerenone is a novel third-generation MRA derived from dihydropyridines, a class distinct from the steroidal backbone of traditional MRAs like spironolactone and eplerenone. This unique structure allows Finerenone to selectively bind to the MR with high affinity while minimizing off-target effects on other steroid receptors, such as androgen and progesterone receptors, which are commonly affected by steroidal MRAs (Figure 3). Quantitatively, the IC50 of Finerenone for the MR is approximately 27.9 nM, compared with 24.2 nM for spironolactone and 990 nM for eplerenone, illustrating its superior binding efficiency [20]. Finerenone’s pharmacokinetic profile is characterized by good oral bioavailability and a predictable, steady drug level that supports once-daily dosing without significant accumulation or prolonged activity from active metabolites, unlike spironolactone [20]. Its binding to the MR is competitive but does not promote the recruitment of transcriptional co-regulators, distinguishing its mechanism of action from traditional steroidal MRAs. This passive antagonism is facilitated by Finerenone’s bulky and rigid molecular structure, which prevents MR activation through specific hydrogen bonding and van der Waals interactions [20]. Its distinct binding characteristics also make Finerenone effective in conditions where MR mutations might render steroidal antagonists less effective, highlighting its potential as a refined third-generation MRA that can better manage risks like hyperkalemia and hormone-related side effects in vulnerable populations [20]. Clinically, the non-steroidal nature of Finerenone offers a targeted and safer option for patients with chronic heart failure and CKD, reducing the likelihood of adverse effects related to steroid receptor cross-reactivity.

### 3.2. Chronic Kidney Disease and Type 2 Diabetes Mellitus

In patients with CKD and T2D, Finerenone has shown promising efficacy and safety in reducing both cardiovascular and kidney risks. Early evidence from the Effect of Finerenone on Albuminuria in Patients With Diabetic Nephropathy (ARTS-DN) trial demonstrated Finerenone’s ability to significantly reduce proteinuria in patients with diabetic nephropathy who were already on ACEi’s or ARBs, with reductions in the urinary albumin–creatinine ratio (UACR) at higher doses [21]. Building on this, the Effect of Finerenone on Chronic Kidney Disease Outcomes in Type 2 Diabetes (FIDELIO-DKD) trial enrolled patients with more advanced CKD stages and showed that Finerenone not only reduced the risk of cardiovascular events by 14% but also slowed kidney disease progression by 18% [22]. These benefits did come with higher rates of hyperkalemia. The Cardiovascular Events with Finerenone in Kidney Disease and Type 2 Diabetes (FIGARO-DKD) trial focused more on cardiovascular protection and earlier CKD stages and showed a significant reduction in heart failure hospitalizations [23]. While the renal benefits were less pronounced in FIGARO-DKD compared with FIDELIO-DKD, there was still a significant reduction in the UACR and lower incidences of end-stage kidney disease. A major criticism was the possibly that some of the patients in these studies were actually benefiting from the sodium–glucose transport protein 2 inhibitor (SGLT2i) and not actually from Finerenone. The pooled FIDELITY analysis, which combined the FIDELIO-DKD and FIGARO-DKD trials, confirmed that Finerenone’s benefits on cardiovascular and kidney outcomes were consistent, whether in the presence or not of an SGLT2i as an adjunct, suggesting an additive protective effect on patient health [24]. A summary of these RCT’s can be seen in Table 1.

### 3.3. Blood Pressure

In 2023, the Effect of Finerenone on Ambulatory Blood Pressure in Chronic Kidney Disease in Type 2 Diabetes (ARTS-DN ABPM) subanalysis explored the effects of Finerenone on ambulatory blood pressure in the ARTS-DN trial. By day 90, Finerenone had led to reductions in the 24 h systolic blood pressure of −8.3 mmHg, −11.2 mmHg, and −9.9 mmHg at doses of 10 mg, 15 mg, and 20 mg, respectively. The study demonstrated consistent blood pressure improvements across all doses, despite Finerenone’s once-daily dosing and short half-life, with no incidents of hypotension reported [25].

### 3.4. Heart Failure

Finerenone has emerged as a promising alternative to traditional MRAs for managing heart failure, particularly because it lacks the unwanted side effects of non-steroidal MRAs. The ARTS study (2013) was the first to evaluate Finerenone in HFrEF patients. It showed that Finerenone at doses of 5 and 10 mg daily reduced biomarkers like BNP, NT-proBNP, and albuminuria as effectively, or better than, spironolactone, with fewer incidents of hyperkalemia and less renal function decline, suggesting a superior safety profile [26]. Compared with eplerenone, considered the safer alternative to spironolactone, Finerenone was similarly effective in reducing NT-proBNP but demonstrated a safer clinical profile, placing it at the forefront of MRAs for safety, as demonstrated in the AA randomized controlled study of finerenone vs. eplerenone in patients with worsening chronic heart failure and diabetes mellitus and/or chronic kidney disease (ARTS-HF) trial [27].

The FIGARO-DKD trial further supported Finerenone’s benefits in patients with chronic kidney disease and type 2 diabetes, showing an 18% reduction in the risk of cardiovascular death or first heart failure hospitalization and a 29% reduction in first hospitalization for heart failure [28]. The FIDELITY-DKD analysis, which pooled the FIGARO-DKD and FIDELIO-DKD studies, confirmed Finerenone’s efficacy in reducing cardiovascular events, particularly heart failure hospitalizations, and showed beneficial kidney outcomes, reinforcing the importance of early treatment in this patient population [29]. Another pooled analysis of these studies further demonstrated Finerenone’s effectiveness in reducing cardiovascular risk, especially for heart failure, across varying severities of chronic kidney disease and levels of albuminuria [30]. Finerenone has thus proven to be a safer and more efficacious alternative to traditional MRAs for HFrEF, but its role in patients with mildly reduced or preserved systolic function remains to be fully explored.

The Finerenone in Heart Failure with Mildly Reduced or Preserved Ejection Fraction (FINEARTS-HF) trial, completed in August 2024, enrolled patients with heart failure with mildly reduced ejection fraction (HFmrEF) and HFpEF. The trial found that Finerenone significantly reduced heart failure events, primarily by decreasing recurrent hospitalizations or urgent visits, although it did not achieve a statistically significant reduction in cardiovascular mortality [31]. A subgroup analysis divided patients between an LVEF < 60% and >60% without distinguishing between preserved and mildly reduced ejection fraction at the 50% cutoff. While 36.5% of patients in the Finerenone group and 36% in the control group had an LVEF < 50%, fitting the mildly reduced group, there was no separate subgroup analysis for this category. The inconsistency in reporting LVEF baselines but not consistently applying these cutoffs to outcomes could lead to misinterpretations. Nonetheless, significant benefits were seen within the <60% group, reinforcing possible positive effects on HFmrEF patients. For those with an LVEF > 60%, no significant difference from placebo was observed in reducing cardiovascular outcomes, suggesting a need for further analyses specifically for HFpEF. Importantly, Finerenone’s benefits were consistent regardless of whether patients were on SGLT2 inhibitors or not, which are strongly recommended in this population.

A large pooled analysis of the FIDELIO-DKD, FIGARO-DKD, and FINEARTS-HF trials demonstrated reductions in heart failure hospitalization and composite kidney outcomes, with a notable reduction in all-cause mortality, although the reduction in cardiovascular death did not reach statistical significance [32]. Overall, Finerenone has shown consistent efficacy and safety across various heart failure phenotypes and comorbid conditions, particularly in reducing heart failure-related events and hospitalizations, establishing its role in the comprehensive management of cardiovascular and renal outcomes in patients with heart failure. A summary of the studies that examined Finerenone in patients with heart failure can be seen in Table 2.

### 3.5. Atrial Fibrillation

Filippatos et al. conducted a secondary analysis of the FIDELIO-DKD trial. Among these, 461 patients had a history of atrial fibrillation (AFib) or flutter. During the study, new-onset AFib occurred in 82 patients treated with Finerenone and 117 patients in the placebo group. The same composite kidney and cardiovascular outcomes were assessed. The analysis revealed that Finerenone’s effect on primary and key secondary kidney and cardiovascular outcomes was not significantly influenced by baseline AFib or flutter. Importantly, Finerenone was associated with a reduced risk of new-onset AFib or flutter, and it demonstrated a consistent reduction in the risk of kidney or cardiovascular disease, regardless of the AFib status at baseline [33].

### 3.6. Ethnicity and Sex Differences

Recent studies have examined the effects of Finerenone on cardiovascular and kidney outcomes across ethnicity and gender. Koya et al. (2023) found that Finerenone lowered cardiovascular event rates in Asians, with 8.6% in the Finerenone group versus 10.0% in the placebo group, and the results were consistent across Asian and non-Asian populations [34]. Bansal et al. (2024) noted that Finerenone’s effect on heart failure hospitalizations was stronger in males, and it reduced kidney events in patients under 75. The drug consistently reduced albuminuria and eGFR decline regardless of sex or age, and gynecomastia was uncommon in males, underscoring its broad efficacy and safety [35].

### 3.7. Head-to-Head Studies with SGLT-2s, GLP-1 RAs, and MRAs

Finerenone’s role in cardiovascular care is emerging through comparisons with traditional MRAs like spironolactone and eplerenone, but comparisons with SGLT2i and glucagon-like peptide-1 receptor agonists (GLP-1 RAs) remain indirect. In HFrEF patients, the ARTS study demonstrated that Finerenone at 5 and 10 mg doses matched or exceeded spironolactone in reducing BNP, NT-proBNP, and albuminuria levels, with fewer incidents of increases in serum potassium and lower rates of hyperkalemia, suggesting a favorable safety profile [26]. When compared with eplerenone in HFrEF patients, the ARTS-HF study found similar reductions in proBNP levels between the two drugs, but also hinted at potential benefits of Finerenone in reducing a composite outcome of death, cardiovascular hospitalizations, or emergency presentations, although this finding was exploratory [27]. Direct head-to-head studies comparing Finerenone with SGLT2i and GLP-1 RAs are lacking. However, a network meta-analysis in patients with T2DM and CKD indicated that SGLT2 inhibitors were superior in reducing renal outcomes and heart failure hospitalizations. While all three drug classes similarly reduced the risk of major adverse cardiovascular events, only SGLT2 inhibitors significantly lowered the cardiovascular death risk, underscoring their superior benefits for renal and heart failure outcomes over Finerenone and GLP-1 RAs [36]. A summary of these studies can be seen in Table 3.

### 3.8. Future Studies

Finerenone’s success will be further tested in several upcoming studies, particularly in managing cardiovascular outcomes and heart failure. The FINALITY-HF, CONFIRMATION-HF, and REDEFINE-HF trials aim to position Finerenone as a potential cornerstone in heart failure treatment, potentially replacing traditional MRAs like spironolactone and eplerenone. REDEFINE-HF builds on the findings of the FINEARTS-HF trial by evaluating cardiovascular outcomes in hospitalized patients with HFpEF and HFmrEF [37]. FINALITY-HF will assess the efficacy and safety of Finerenone in patients with heart failure and reduced ejection fraction who are intolerant of or ineligible for steroidal MRAs, potentially establishing it as not just an alternative but a first-line MRA in heart failure treatment [38].

The CONFIDENCE study serves as a strong precursor to CONFIRMATION-HF by exploring the combined effects of Finerenone and empagliflozin on albuminuria reduction in CKD patients with type 2 diabetes, underscoring the potential of combination therapies to enhance both renal and cardiovascular outcomes [39]. CONFIRMATION-HF will further this research by investigating Finerenone combined with SGLT2 inhibitors in a broader HF population, regardless of the LVEF, with the goal of reducing heart failure events and cardiovascular mortality [40]. This could help cement Finerenone’s role as an adjunct to current guideline-directed medical therapies in heart failure.

The FIVE-STAR study contributes to the cardiovascular narrative by focusing on vascular stiffness and cardiorenal biomarkers in patients with type 2 diabetes and CKD, highlighting Finerenone’s potential impact on vascular health [41]. Meanwhile, the FIONA and FINE-ONE studies, though more niche, demonstrate Finerenone’s expanding utility beyond traditional adult populations. FIONA explores its efficacy in pediatric patients with CKD and proteinuria, marking Finerenone’s entry into younger demographics [42]. FIONA-OLE is an extension study of FIONA that will assess the long-term safety (18 months) of Finerenone in the same cohort [42]. Similarly, FINE-ONE investigates its role in type 1 diabetes, indicating Finerenone’s growing application in managing conditions beyond CKD and heart failure [43]. FIND-CKD will look to assess Finerenone’s effect on cardiorenal outcomes in patients with CKD but without diabetes [44].

Collectively, these studies intend to propel Finerenone’s expanding role in cardiovascular care, not only in CKD and diabetes but also in heart failure (Table 4).

## 4. Discussion

Finerenone is a non-steroidal, selective antagonist of the mineralocorticoid receptor specifically developed to counteract the harmful effects of MR overactivation. Its targeted action on the MR distinguishes it from traditional MRAs like spironolactone and eplerenone, which tend to have off-target effects in varying degrees. It reduces cardiovascular damage linked to excessive MR activation while minimizing side effects associated with androgen and progesterone receptor interactions. Currently approved in over 90 countries for the treatment of CKD associated with T2DM, Finerenone’s broader therapeutic potential is being explored through the FINEOVATE research program, which includes several Phase III trials discussed in this review.

Finerenone has shown significant cardiovascular and renal benefits in patients with CKD and T2D, making it a promising alternative to existing MRAs. Current guidelines from the American College of Cardiology (ACC) and the European Society of Cardiology (ESC) recommend MRAs like spironolactone and eplerenone for patients with HFrEF. However, as evidence supporting Finerenone’s broader efficacy and safety in CKD, diabetes, and heart failure continues to grow, these guidelines may evolve to include Finerenone, especially for patients who are intolerant of or less responsive to traditional steroidal MRAs. As HF management continues to evolve, especially for patients with CKD and T2DM, the current guidelines may not fully address the challenges these patients face. Finerenone’s effectiveness and better safety profile in these groups highlight the need for updated guidelines that include newer treatment options, which could improve outcomes for these high-risk patients. Finerenone may also find a unique role in managing HFmrEF and HFpEF, areas where traditional MRAs have limited impact.

In clinical trials, Finerenone has demonstrated similar or superior efficacy in reducing biomarkers like NT-proBNP compared with spironolactone and eplerenone, and it shows fewer adverse effects. Beyond its impact on cardiorenal outcomes, Finerenone has also been shown to effectively lower blood pressure in patients with CKD and T2D, with significant reductions in systolic blood pressure reported in the ARTS-DN trial. Additionally, secondary analyses from the FIGARO-DKD trial suggest that Finerenone may reduce the incidence of new-onset atrial fibrillation, setting it apart from other MRAs. Finerenone’s ability to reduce proteinuria and slow the progression of CKD further establishes its role as an essential therapeutic option in the management of diabetes, complementing its cardiovascular benefits in heart failure patients.

Finerenone’s safety profile is another key advantage, as it is associated with a lower incidence of hyperkalemia compared with spironolactone and demonstrates a more favorable renal safety profile, with fewer instances of acute kidney injury (AKI). Unlike steroidal MRAs, Finerenone does not cause anti-androgenic side effects such as gynecomastia, impotence, and menstrual irregularities. The ARTS-HF study further supports Finerenone’s safety, showing fewer increases in serum potassium and less deterioration of renal function compared with eplerenone, making it an even safer alternative for patients who cannot tolerate spironolactone.

In the broader context of heart failure treatment, Finerenone’s role among established therapies remains under investigation. While SGLT2 inhibitors and GLP-1 RAs have demonstrated significant cardiovascular and renal benefits in CKD and T2D populations, network meta-analyses indicate that SGLT2 inhibitors outperform both Finerenone and GLP-1 RAs in reducing renal outcomes and heart failure hospitalizations [34]. Although all three classes reduce major adverse cardiovascular events, only SGLT2 inhibitors have shown a significant impact in reducing cardiovascular death. Finerenone may find its niche in combination therapy, as ongoing trials like CONFIDENCE and CONFIRMATION explore the potential synergistic effects of combining Finerenone with SGLT2 inhibitors, potentially broadening its therapeutic applications and enhancing patient outcomes.

This positioning of Finerenone reflects its evolving role in comprehensive cardiovascular and renal management, with future studies poised to further clarify its standing alongside, or in conjunction with, other established therapies. We provided a table that summarizes the comparisons of Finerenone to steroidal MRAs with respect to various characteristics and side effects (Table 5).

## 5. Conclusions

Finerenone represents a significant advancement in the management of heart failure, CKD, and T2D, offering a clearly better side-effect profile compared with traditional MRAs, especially in HFrEF, where it demonstrates superior safety and potential efficacy. While the early data suggest possible benefits in patients with HFmrEF, its role in HFpEF remains less certain and requires further investigation. More randomized controlled trials and subgroup analyses, particularly from the FINEART-HF trial, are necessary to better assess outcomes in specific groups. Additionally, new RCTs that focus specifically on these populations, ideally in a head-to-head format, are essential to solidify Finerenone’s position in cardiovascular and renal care and to determine its full therapeutic potential across different heart failure phenotypes.

## Figures and Tables

**Table 1:** Summary of RCT’s on the efficacy and safety of Finerenone in patients with CKD and T2DM.

Study (Year)	Design	Intervention	Control	Main Outcomes	Effect Size
ARTS-DN (2015) [21]	RCT	Finerenone 1.25–20 mg once daily	Placebo	UACR over 90 days	There were reductions in UACR across all doses of Finerenone
FIDELIO-DKD (2020) [22]	RCT	Finerenone 10 or 20 mg once daily	Placebo	Kidney composite	Finerenone reduced the risk of CKD progression by 18%
FIGARO-DKD (2021) [23]	RCT	Finerenone 10 or 20 mg once daily	Placebo	Cardiovascular composite	Finerenone reduced the risk of composite CV events by 13%
The FIDELITY analysis (2022) [24]	Pooled analysis of FIDELIO-DKD and FIGARO-DKD	Finerenone 10 or 20 mg once daily	Finerenone + SGLT2i	Cardiovascular composite and kidney composite	Finerenone without an SGLT2i was equally as effective in reducing cardiorenal outcomes as Finerenone with SGLT2i

RCT = randomized controlled trial; SGLT2i = sodium–glucose transport protein 2 inhibitor; UACR = urine albumin–creatinine ratio; CKD = chronic kidney disease; CV = cardiovascular.

**Table 2:** Summary of randomized controlled trials of Finerenone on heart failure.

Study (Year)	Design	Population	Intervention vs. Control	Primary Outcome	Result
ARTS part A and B (2013) [26]	RCT	HFrEF and CKD	Finerenone 2.5–10 mg once daily vs. placebo (part A) and spironolactone (part B)	Change in serum potassium	Finerenone 10 mg once daily and 5 mg twice daily led to higher mean increases in serum potassium than the placebo but lower levels than spironolactone.
ARTS-HF (2016) [27]	RCT	HFrEF and T2D and/or CKD	Finerenone 2.5–15 mg once daily vs. eplerenone 25–50 mg once daily	>30% decline in NT-proBNP	There was no difference in NT-proBNP decline in Finerenone versus eplerenone.
Filippatos et al., 2022 [28]	Post hoc		Finerenone 10 or 20 mg once daily vs. placebo	First HHF, the combined risk of cardiovascular death or first HHF, and the risk of new-onset heart failure.	Finerenone had a 29% reduction in first HHF, 18% in cardiovascular death or first HHF, and 32% in new-onset HF.
Agarwal et al. (2022) [29]	Post hoc	CKD and T2D	Finerenone 10 or 20 mg once daily vs. placebo	Cardiovascular and renal outcomes	Finerenone reduced the risk of cardiovascular and kidney outcomes vs. placebo across the spectrum of CKD in patients with type 2 diabetes.
Agarwal et al. (2023) [30]	Post hoc	CKD and T2D	Finerenone 10 or 20 mg once daily vs. placebo	Incidence rates of cardiovascular events	Finerenone was associated with a reduction in composite cardiovascular risk irrespective of eGFR and UACR.
FINEARTS-HF (2024) [31]	RCT	HFmrEF and HFpEF	Finerenone 20 or 40 mg once daily vs. placebo	Composite of total worsening heart failure events and death from cardiovascular causes	Finerenone resulted in a lower rate of total worsening heart failure events and death from cardiovascular causes than the placebo.
FINE-HEART (2024) [32]	Pooled analysis of FIGARO-DKD, FIDELIO-DKD, and FINEARTS-HF trials	-	Finerenone 20 or 40 mg once daily vs. placebo	Cardiorenal outcomes	Failed to demonstrate significant reductions in cardiovascular death; however, Finerenone was associated with significantly lower deaths of any cause, cardiovascular events, and kidney outcomes

RCT = randomized controlled trial; HFrEF = heart failure with reduced ejection fraction; CKD = chronic kidney disease; T2D = type 2 diabetes; NT-proBNP = N-terminal pro B-type natriuretic peptide; HHF = hospitalization for heart failure; HF = heart failure; HFmrEF = heart failure with mildly reduced ejection fraction; HFpEF = heart failure with preserved ejection fraction; eGFR = estimated glomerular filtration rate; UACR = urine albumin-to-creatinine ratio.

**Table 3:** Summary of Finerenone in head-to-head randomized controlled trials.

Study (Year)	Design	Population	Intervention vs. Control	Primary Outcome	Effect Size
Zhang et al. (2022) [36]	Network meta-analysis	-	Finerenone vs. SGLT2i and GLP-1 agonist	Cardiorenal outcomes	SGLT2i significantly decreased the risk of renal events and HHF in comparison. All three were comparable in MACE, ACD, and CVD.
ARTS part A and B (2013) [26]	RCT	HFrEF and CKD	Finerenone 2.5–10 mg once daily vs. placebo (part A) and spironolactone (part B)	Change in potassium	Finerenone 10 mg once daily and 5 mg twice daily led to higher mean increases in serum potassium than the placebo but lower levels than spironolactone
ARTS-HF (2016) [27]	RCT	HFrEF and T2D and/or CKD	Finerenone 2.5–15 mg once daily vs. eplerenone 25–50 mg once daily	>30% decline in NT-proBNP	There was no difference in the NT-proBNP decline in Finerenone versus eplerenone

RCT = randomized controlled trial; HFrEF = heart failure with reduced ejection fraction; CKD = chronic kidney disease; T2D = type 2 diabetes; SGLT2i = sodium–glucose cotransporter-2 inhibitor; GLP-1 agonist = glucagon-like peptide-1 receptor agonist; NT-proBNP = N-terminal prohormone of brain natriuretic peptide; HHF = hospitalization for heart failure; MACE = major adverse cardiovascular events; ACD = all-cause death; CVD = cardiovascular death.

**Table 4:** Anticipated randomized controlled trials.

Study	Design	Anticipated Completion Date	Population	Intervention vs. Control	Primary Outcome
REDEFINE-HF [37]	RCT	April 2026	Adults with heart failure with an EF > 40%	Finerenone vs. placebo	Composite total of HF events and CV death
FINALITY-HF [38]	RCT	August 2026	HFrEF and intolerant of non steroidal MRAs	Finerenone vs. placebo	Composite total of HF events and CV death
CONFIDENCE [39]	RCT	January 2025	Patients with CKD and T2DM on ACEis or ARBs	Finerenone + empagliflozin vs. placebo + Finerenone or empagliflozin	Urinary albumin to-creatinine ratio (UACR)
CONFIRMATION-HF [40]	RCT	November 2025	Adults with heart failure	Finerenone + empagliflozin vs. standard of care	Clinical benefit
FIVE-STAR [41]	RCT	July 2026	CKD and T2DM	Finerenone vs. placebo	Change in CAVI (Cardio–ankle vascular index) at 24 weeks after initiation of the protocol treatment compared with baseline
FIONA [42]	RCT	March 2027	Children (6 months to 18 years old)	Finerenone + ACEi/ARB vs. placebo	Urinary protein-to-creatinine ratio (UPCR) reduction of at least 30% from baseline to day 180
FIONA-OLE [42]	RCT	18-month extension of FIONA	Children (6 months to 18 years old)	Finerenone + ACEi/ARB vs. placebo	Long-term safety
FINE-ONE [43]	RCT	October 2025	Adults with T1DM	Finerenone vs. placebo	Change in UACR
FIND-CKD [44]	RCT	February 2026	CKD without diabetes	Finerenone vs. placebo	Change in eGFR from baseline to 32 months

RCT = randomized controlled trial; EF = ejection fraction; HF = heart failure; CV = cardiovascular; HFrEF = heart failure with reduced ejection fraction; MRA = mineralocorticoid receptor antagonist; CKD = chronic kidney disease; T2DM = type 2 diabetes mellitus; ACEi = angiotensin-converting enzyme inhibitor; ARB = angiotensin II receptor blocker; UACR = urinary albumin-to-creatinine ratio; CAVI = cardio–ankle vascular index; UPCR = urinary protein-to-creatinine ratio; T1DM = type 1 diabetes mellitus; eGFR = estimated glomerular filtration rate.

**Table 5:** Comparison of the characteristics and effects of MRAs.

	Steroidal MRA		Non-Steroidal MRA
Characteristics	Spironolactone	Eplerenone	Finerenone
Antagonism over MR	High	Low	High
Antagonism over AR	High	Low	Low
Effect on proteinuria and kidney damage	Moderate	Low	High
BP reduction	High	Low	High
Effect on HFpEF	Moderate	Moderate	High
Effect on HFmrEF	Moderate	Moderate	High
Effect on HFrEF	High	High	High
Hyperkalemia	High	Moderate	Low
Gynecomastia	High	Moderate	Low

MRA = mineralocorticoid receptor antagonist; MR = mineralocorticoid receptor; AR = androgen receptor; BP = blood pressure; HFpEF = heart failure with preserved ejection fraction; HFmrEF = heart failure with moderately reduced ejection fraction; HFrEF = heart failure with reduced ejection fraction.

## Data Availability

In this scoping review, all data used were sourced from publicly accessible online databases, and no new data were generated. Consequently, no additional materials, data collection forms, or analytic code are available beyond what is already publicly accessible. All referenced data can be accessed directly through the original sources as cited in the review.
